# Serum Vitamin D Levels, Systemic Inflammation, and Exacerbation Among Patients with COPD GOLD Group E

**DOI:** 10.3390/biomedicines14040833

**Published:** 2026-04-06

**Authors:** Apostolos Sioutas, Hans Lennart Persson

**Affiliations:** 1 Department of Oncology in Linköping, Department of Health, Medicine and Caring Sciences, Linköping University, 581 83 Linkoping, Sweden; 2 Department of Respiratory Medicine in Linköping, Department of Health, Medicine and Caring Sciences, Linköping University, 581 83 Linkoping, Sweden; lennart.persson@liu.se

**Keywords:** chronic obstructive pulmonary disease (COPD), systemic inflammation, exacerbations, vitamin D deficiency, serum 25-hydroxyvitamin D (25(OH)D), GOLD group E, inflammatory markers, high-sensitivity C-reactive protein (hs-CRP), COPD symptom burden, lung function

## Abstract

**Background**: Chronic obstructive pulmonary disease (COPD) is associated with systemic inflammation and frequent exacerbations, leading to disease progression and increased morbidity. Vitamin D deficiency has been suggested to contribute to COPD inflammation and exacerbations. **Aim**: This study investigated the association between serum 25-hydroxyvitamin D (25(OH)D) levels, systemic inflammation, and exacerbation frequency among patients with COPD GOLD group E. **Methods**: A cross-sectional study was conducted on 111 patients with stable COPD. Patients were divided into two groups based on their serum 25(OH)D levels (<50 nmol/L vs. ≥50 nmol/L). Data on exacerbation frequency for the past year, inflammatory markers, spirometric lung function parameters, and symptom burden were collected. **Results**: Patients with low serum 25(OH)D (<50 nmol/L) had a significantly higher CAT score and level of serum high-sensitivity (hs)-CRP and exhibited significantly more exacerbations compared to those with higher 25(OH)D levels (*p* < 0.001, *p* < 0.001, and *p* < 0.0001, respectively). Furthermore, lower vitamin D levels were associated with higher CAT scores (Pearson’s correlation coefficient, r = −0.30, *p* < 0.01) and higher serum hs-CRP levels (Spearman’s rank correlation coefficient, r = −0.25, *p* < 0.01), as well as a higher number of exacerbations (Pearson’s correlation coefficient, r = −0.74, *p* < 0.0001). **Conclusions**: Low vitamin D levels are significantly associated with greater symptom burden, elevated hs-CRP, and increased exacerbation frequency, indicating a strong relationship between vitamin D deficiency, systemic inflammation, and disease burden in patients with COPD belonging to GOLD group E. However, due to the cross-sectional design, no causal relationship can be inferred and prospective interventional studies are required to determine whether treating vitamin D deficiency improves clinical outcomes.

## 1. Introduction

Due to Sweden’s northern latitude and extended periods of low sunlight, maintaining adequate vitamin D synthesis can be challenging [1]. In addition, for patients with severe chronic obstructive pulmonary disease (COPD), physical limitations often reduce outdoor activity [2,3]. Among elderly patients with advanced COPD, previous research has suggested that 50–70% may display vitamin D deficiency, i.e., serum 25-hydroxyvitamin D [25(OH)D] levels below 50 nmol/L [4,5,6,7]. Since 25(OH)D is the stable circulating metabolite of vitamin D, it is the preferred marker for serum analysis.

The relationship between vitamin D deficiency and COPD progression remains unclear [8,9,10]. It is possible that a lack of vitamin D merely reflects generally poor health rather than being a direct cause of COPD progression [11]. However, evidence suggests that vitamin D may have anti-inflammatory effects that could counteract the inflammatory processes involved in COPD [12]. Thus, low serum 25(OH)D levels are associated with poor lung function [13,14,15], increased respiratory symptoms [7,16,17], and reduced quality of life [7,16,18].

Given the known anti-inflammatory effects of vitamin D [12], it is reasonable to investigate its role in COPD exacerbations. Multiple studies have also reported a link between low serum 25(OH)D levels and an increased frequency of COPD exacerbations [19,20,21,22,23,24,25]. Two recent studies from 2021 examined the relationship between serum 25(OH)D and systemic inflammation in COPD patients and demonstrated that the strongest correlation was with the inflammatory marker C-reactive protein (CRP) [26,27]. However, the correlations of these two studies were rather weak (Spearman; r = −0.25, *p* < 0.01 and r = −0.32, *p* < 0.01, respectively), which might be explained by the mixed COPD stages (I–IV) that were included [26,27]. Thus, to better understand the role of low serum 25(OH)D levels in systemic inflammation and COPD exacerbations, we hypothesized that patients with COPD and frequent exacerbations, i.e., GOLD group E, would be more relevant for further studies.

Therefore, the objectives of the present study were to investigate patients with COPD belonging to GOLD group E, to better understand whether low serum 25(OH)D is associated with raised levels in serum of markers of systemic inflammation and to study the relationships between serum 25(OH)D and other clinical variables such as symptom burden and COPD exacerbation frequency over the past year.

## 2. Methods

### 2.1. Study Design and Data Collection

The present study was a monocentric cross-sectional observational study conducted at the Department of Respiratory Medicine in Linköping, Sweden. Patients were recruited consecutively between August 2012 and October 2018.

Eligible participants were adults aged >45 years with a confirmed diagnosis of COPD, defined by persistent airflow limitation with post-bronchodilator forced expiratory volume in one second (FEV_1_)/forced vital capacity (FVC) < 0.70, in accordance with GOLD recommendations. All patients belonged to GOLD group E and had a clinically stable phase of the disease.

Exclusion criteria comprised ongoing supplementation with calcium or vitamin D, COPD exacerbation or respiratory infection within two weeks prior to inclusion, ongoing exacerbation at the time of evaluation, systemic inflammatory disease, cognitive impairment, unwillingness to participate, or language barriers preventing informed consent.

At inclusion, data were collected on demographic characteristics (age, sex), smoking status, body mass index (BMI), COPD medication use, symptom burden assessed by the COPD Assessment Test (CAT) [28], dyspnea severity using the modified Medical Research Council scale (mMRC) [29], and comorbidities assessed by the Charlson Comorbidity Index (CCI) [30]. Exacerbation frequency during the preceding 12 months (moderate and severe exacerbations only) was also recorded.

### 2.2. Data Handling and Data Protection

All collected data were pseudonymized and handled according to the General Data Protection Regulation (GDPR). Data were securely stored and accessible only to authorized study personnel.

### 2.3. Patient and Public Involvement

Patients and the public were not involved in the development of the study design, data collection procedures, analysis, or manuscript preparation. The study results were not disseminated directly to participants.

### 2.4. Determining Concentrations of Vitamin D Hydroxy Metabolites

Venous peripheral blood samples were collected at study inclusion during a clinically stable phase of COPD in the absence of acute exacerbation, and centrifuged. Blood samples were collected between August 2012 and October 2018; however, the exact month of sampling for individual participants was not systematically documented for analytical purposes. Plasma was transferred to microtubes and stored at −80 °C until analysis. Plasma concentrations of 25-hydroxyvitamin D_2_ and D_3_ were determined using high-performance liquid chromatography coupled with electrospray tandem mass spectrometry (HPLC–ESI-MS/MS), (TSQ Quantum Access Max, Thermo Fisher Scientific, Waltham, MA, USA) following the method described by Turpeinen et al. [31]. Samples were derivatized with 4-phenyl-1,2,4-triazoline-3,5-dione (Sigma-Aldrich, St. Louis, MO, USA) prior to analysis. Quality assurance was ensured through participation in the Vitamin D External Quality Assessment Scheme (DEQAS) [7]. All analyses were performed at the Division of Occupational and Environmental Medicine, Linköping University, Sweden.

### 2.5. Oxygenation, Inflammatory Biomarkers, Spirometry, and GOLD-Based Chronic Obstructive Pulmonary Disease Staging

Blood oxygenation was assessed by measuring peripheral oxygen saturation (SAT) at rest. Venous blood samples were collected at inclusion to assess systemic inflammatory markers including white blood cell count (WBC), and high-sensitivity C-reactive protein (hs-CRP) using routine clinical laboratory methods. All hs-CRP analyses were performed in the same certified clinical laboratory using standardized methods [32]

Spirometry was performed using a Jaeger spirometer (MasterScreen^TM^ PFT System, CareFusion, Hoechberg, Germany) both before and after bronchodilator administration with salbutamol (Ventoline^®^, GlaxoSmithKline, Brentford, UK). Post-bronchodilator measurements were obtained following inhalation of the short-acting bronchodilator salbutamol (0.1 mg per dose × four doses) administered via a spacer device (Volumatic^®^, GlaxoSmithKline, Brentford, UK). Lung function results are expressed as percentages of predicted values using the Hedenström reference equations [33,34].

COPD severity was classified according to the degree of airflow obstruction based on post-bronchodilator forced expiratory volume in one second (FEV_1_ % predicted) with staging defined as: GOLD stage I (80–100%), stage II (50–79%), stage III (30–49%), and stage IV (<30%). All included patients fulfilled the criteria for GOLD group E, indicating a history of frequent exacerbations.

### 2.6. Statistical Analysis

Continuous variables are presented as mean ± standard deviation (SD) when normally distributed and as median with interquartile range (IQR) when normality assumptions were not fulfilled. Categorical variables are expressed as absolute numbers and percentages. Patients were grouped according to serum 25-hydroxyvitamin D 25(OH)D levels (<50 nmol/L vs. ≥50 nmol/L).

Associations between serum 25(OH)D levels and clinical, functional, and inflammatory variables were assessed using Pearson’s correlation coefficient for normally distributed data and Spearman’s rank correlation coefficient for non-normally distributed data. All statistical tests were two-tailed, and a *p*-value < 0.05 was considered statistically significant. Statistical analyses were performed using IBM SPSS Statistics, version 27.0 (IBM Corp., Chicago, IL, USA).

## 3. Results

### 3.1. Characterization of the Study Population

One hundred eleven patients with COPD, GOLD group E were included in the data analysis from August 2012 to October 2018 and divided into two groups: those with serum 25(OH)D < 50 nmol/L (hereafter called “low”) and those with serum 25(OH)D ≥ 50 nmol/L (hereafter called “high”). Variables without significant difference are summarized in Table 1. The two groups did not differ significantly regarding gender, age, smoking status, BMI, mMRC, CCI, WBC, SAT, post-bronchodilator FEV_1_ as % of predicted, GOLD stage, and ongoing medications for COPD, including long-term oxygen therapy (LTOT). None of the patients were receiving calcium or vitamin D supplementation. Since all included patients belonged to GOLD group E and were treated according to contemporary guidelines, no significant differences in maintenance pharmacotherapy were observed between vitamin D groups.

### 3.2. Vitamin D Status Has a Significant Impact on CAT, hs-CRP, and Exacerbation Frequency

**Figure 1:**
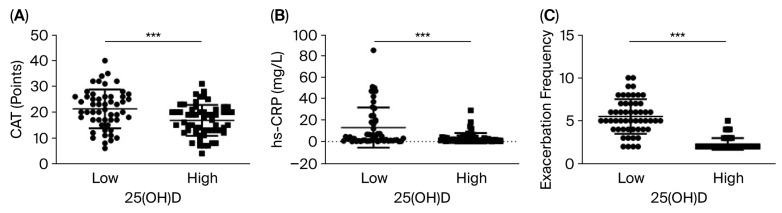
Differences in symptom burden, systemic inflammation, and exacerbation frequency between COPD patients with low and high serum 25(OH)D levels. (**A**) CAT score, (**B**) hs-CRP, (**C**) exacerbation frequency in COPD GOLD Group E, ***: *p* < 0.001.

The group with patients displaying low serum 25(OH)D had significantly higher CAT scores and levels of serum hs-CRP and exhibited significantly more exacerbation incidents compared to those with a high level of serum 25(OH)D (*p* < 0.001, *p* < 0.001, and *p* < 0.0001, respectively). The results are shown in Figure 1.

Median hs-CRP was 4.7 mg/L (IQR 1.1–19.0) in the low-vitamin-D group compared to 1.7 mg/L (IQR 0.8–3.3) in the high-vitamin-D group. The median number of exacerbation incidents during the preceding year was five (IQR 4–7) versus two (IQR 2–3), respectively.

### 3.3. Vitamin D Level Correlates Significantly with CAT, hs-CRP, and Exacerbation Frequency

**Figure 2:**
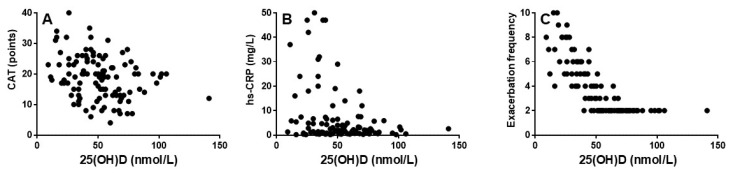
Correlations between serum 25(OH)D levels and (**A**) CAT score, (**B**) hs-CRP, and (**C**) exacerbation frequency in COPD GOLD Group E.

Low serum 25(OH)D was associated with high CAT scores (r = −0.30; *p* < 0.01) and levels of serum hs-CRP (Spearman; r = −0.25; *p* < 0.01), as well as significantly more exacerbations during the past year (r = −0.74; *p* < 0.0001) compared to those with high serum 25(OH)D. The results are shown in Figure 2. No significant associations were observed between serum 25(OH) and the other study variables presented in Table 1.

## 4. Discussion

In this cross-sectional study of 111 patients with COPD, group GOLD E without ongoing vitamin D supplementation, low serum 25(OH)D was associated with greater COPD symptom burden (shown as a high CAT score), an elevated level of hs-CRP, and an increased exacerbation frequency during the past year (Figure 1). To the best of our knowledge, the present study is the first to show in the same study, that symptom burden, systemic inflammation, and exacerbation frequency are all associated with serum 25(OH)D levels (Figure 2). No significant associations were observed between serum 25(OH)D and the other study variables. These findings reinforce the idea that vitamin D plays a role in immune regulation and inflammation related to COPD.

Recent publications from 2024 to 2025 further support the association between vitamin D deficiency and increased COPD exacerbations [35,36]. However, evidence regarding causality and therapeutic benefits remains conflicting, as a recent Cochrane review failed to demonstrate consistent protective effects of vitamin D supplementation [37]. Severe vitamin D deficiency, however, was strongly associated with hospitalization for COPD exacerbation in the previous year [25]. In contrast to previous heterogeneous COPD populations, the present study exclusively investigated patients classified as GOLD group E.

In a previous study of a cohort of patients with severe COPD, our research team showed that ongoing vitamin D supplementation was the most important intervention to maintain serum 25(OH)D levels above 50 nmol/L [7]. In the present study, none of the patients included were supplemented with vitamin D. Compared with earlier studies demonstrating a 50–70% prevalence of vitamin D deficiency among patients with COPD [4,5,6,7], a more recent Swedish study reported only 33% of study subjects demonstrating vitamin D deficiency [38]. In that study, subjects were included from January 2017 to December 2018 and from January to December 2021 [38].

The Swedish regulation on food fortification changed in 2018 to include more products and higher fortification levels of vitamin D [39]. As a result, more of the population can reach the recommended intake of vitamin D from food. In recent years, increased awareness of osteoporosis risk among the elderly has led to routine prescriptions of calcium and vitamin D supplements. In addition, due to increased public awareness among the elderly that vitamin D deficiency is associated with an increased risk of respiratory infections [40], purchases of over-the-counter supplements containing vitamin D have increased.

Recruitment challenges in vitamin D supplementation trials have been illustrated in the PRECOVID study, where a large proportion of screened patients were excluded due to ongoing supplementation or sufficient baseline vitamin D levels [41]. Such factors may reduce statistical power and limit the ability to detect a treatment effect. These findings highlight the methodological complexity of conducting adequately powered randomized controlled trials (RCTs) in contemporary populations with a relatively lower prevalence of severe vitamin D deficiency. In the present study, all subjects were recruited from August 2012 to October 2018, when vitamin D deficiency still had a high prevalence among Swedish patients with COPD.

Numerous previous studies have shown that adequate vitamin D levels were associated with a lower burden of COPD symptoms [7,16,17]. The result of the present study is in line with these studies, as it shows a significant negative correlation between serum-25(OH)D and CAT score (r = −0.30, *p* < 0.01) (Figure 2A).

To the best of our knowledge, there is only one previous study similar to the present one [27]. Jorde et al. recruited study subjects during 2011–2012 and demonstrated a significant association between low levels of serum 25(OH)D and increased levels of CRP [27]. The correlation was rather weak (Spearman; r = −0.32, *p* < 0.01; *n* = 94) [27]. In another study, the same correlation was even weaker (Spearman; r = −0.25, *p* < 0.01; *n* = 101) [26]. These observations gave us the idea to test whether this correlation could be strengthened by a study on only frequent exacerbators (*n* = 111) and by employing high-sensitivity CRP to achieve a more fine-tuned assessment of systemic inflammation. In contrast to what we expected, these alterations of the study conditions produced almost the same result (Spearman; r = −0.25, *p* < 0.01) (Figure 2B). We conclude that the present study provides additional evidence for an association in patients with COPD between vitamin D deficiency and systemic inflammation and past exacerbations (Figure 2B,C).

In the present study, we also analyzed WBC and fibrin as they are well-known markers of systemic inflammation. None of these markers demonstrated a significant difference when patients with low serum-25(OH)D were compared with patients exhibiting high level of serum-25(OH)D (Table 1). We decided to not complete the analysis of Interleukin-6 (IL-6) in all subjects, since Jorde et al. [27] already demonstrated that IL-6 displayed a weaker correlation (Spearman; r = −0.23, *p* = 0.028) with serum 25(OH)D than CRP did.

Unlike Jorde et al. [27], who did not find a correlation between the serum 25(OH)D level and the number of exacerbations during the past year, we found a very strong correlation (r = −0.74, *p* < 0.0001). This difference was probably due to the selection of patients with frequent exacerbation incidents. This observation is in line with previous studies [19,20,21,22,23,24,25], but unlike these studies, the present study assesses the important link between systemic inflammation and serum 25(OH)D.

The exact mechanisms by which vitamin D deficiency influences the inflammatory process observed in COPD remain incompletely understood. Notably, the first report on a link between vitamin D deficiency and iron-mediated oxidative damage in the pathogenesis of COPD was recently published [42]. In this context, vitamin D deficiency was positively associated with glutathione peroxidase 4 reduction and iron parameter elevation in COPD patients [42]. Many studies support the crucial role of transition metals such as ferrous iron and oxidative damage on lung cells as the cause behind the structural changes observed in the airways and lung tissues of patients with COPD [42,43,44,45,46].

Recent studies suggest that vitamin D deficiency may also impair airway epithelial hydration. Calcitriol deficiency increases epithelial sodium channel (ENaC) activity, enhancing sodium and water absorption from the airway surface liquid. Reduced surface hydration may compromise mucociliary clearance and innate immune defense, promoting mucus plugging and increasing susceptibility to exacerbations [47].

## 5. Limitations

Taken together with the recent literature, the present study does not aim to establish causality but rather to reinforce the robustness of the association between severe vitamin D deficiency, systemic inflammation, and exacerbation burden in a well-defined North-European high-risk COPD population.

Several limitations of the present study should be acknowledged. First, the cross-sectional design precludes conclusions regarding causality between serum 25(OH)D levels, systemic inflammation, and exacerbation frequency. Second, seasonal variation in vitamin D levels was not accounted for. Blood samples were collected over an extended period (August 2012 to October 2018) and the exact timing of plasma collection in relation to season or sunlight exposure was not controlled for, which may have influenced measured 25(OH)D concentrations. Third, although patients receiving vitamin D supplementation were excluded, dietary vitamin D intake, sun exposure habits, and outdoor physical activity were not assessed and may have acted as residual confounding factors. Fourth, systemic inflammation was primarily evaluated using hs-CRP and WBC, while other inflammatory biomarkers were not systematically analyzed. Fifth, the single-center design and inclusion of only patients belonging to the GOLD group E may limit the generalizability of the findings to patients with less severe COPD or other GOLD categories. Finally, vitamin D deficiency may represent a marker of reduced outdoor activity, nutritional habits, or socio-economic determinants rather than a direct mechanistic driver. Despite strong associative evidence, the most recent Cochrane review (2024) concluded that vitamin D supplementation does not consistently prevent acute COPD exacerbations [37].

## 6. Conclusions

The results of the present study demonstrate a strong association between low vitamin D levels, systemic inflammation, and exacerbation frequency in COPD patients belonging to GOLD group E. However, prospective interventional studies are required to clarify whether treating vitamin D deficiency improves clinical outcomes.

## Figures and Tables

**Table 1:** Baseline characteristics of COPD patients by serum 25(OH)D level (<50 vs. ≥50 nmol/L).

COPD Patients *n* = 111	Low (<50 nmol/L) *n* = 53	High (≥50 nmol/L) *n* = 58	Difference
Age (yrs)	70 ± 10	70 ± 7	n.s.
Women, *n* (%)	28 (53)	34 (57)	n.s.
BMI (kg/m^2^)	26 ± 7	25 ± 5	n.s.
Current smokers, *n* (%)	12 (23)	7 (12)	n.s.
Ex smokers, *n* (%)	39 (74)	49 (84)	n.s.
Never smokers, *n* (%)	2 (4)	2 (3)	n.s.
mMRC (points)	3 (1)	3 (1)	n.s.
CCI, score 1, *n* (%)	12 (23)	18 (31)	n.s.
CCI, score 2, *n* (%)	13 (25)	7 (12)	n.s.
CCI, score 3, *n* (%)	14 (26)	14 (24)	n.s.
CCI, score ≥ 4, *n* (%)	14 (26)	19 (33)	n.s.
WBC	9.8 ± 4.2	8.7 ± 2.3	n.s.
SAT (%)	92 ± 6	94 ± 4	n.s.
FEV_1_ (% predicted)	42 ± 17	41 ± 17	n.s.
GOLD stage 1, *n* (%)	1 (2)	0 (0)	n.s.
GOLD stage 2, *n* (%)	18 (34)	17 (29)	n.s.
GOLD stage 3, *n* (%)	14 (26)	22 (38)	n.s.
GOLD stage 4, *n* (%)	20 (38)	18 (31)	n.s.
*COPD medications*			
SABA, *n* (%)	39 (74)	48 (83)	n.s.
SAMA, *n* (%)	15 (28)	14 (24)	n.s.
LABA, *n* (%)	51 (96)	56 (97)	n.s.
LAMA, *n* (%)	50 (94)	53 (91)	n.s.
ICS, *n* (%)	50 (94)	54 (93)	n.s.
OCS, *n* (%)	13 (25)	13 (22)	n.s.
PD4I, *n* (%)	7 (13)	7 (12)	n.s.
LTOT, *n* (%)	16 (30)	12 (21)	n.s.

Abbreviations: BMI, body mass index; CCI, Charlson Comorbidity Index; COPD, chronic obstructive pulmonary disease; FEV_1_ (% of predicted), forced expiratory volume in one second expressed as % of predicted; GOLD, global initiative for chronic obstructive lung disease; ICS, inhaled corticosteroids; LABA, long-acting beta-2 agonists; LAMA, long-acting muscarin antagonist; LTOT, long-term oxygen therapy; mMRC, modified Medical Research Council dyspnoea scale; n.s., not significant; PD4I, phosphodiesteras 4 inhibitor; OCS, oral corticosteroids; SABA, short-acting beta-2-agonists; SAMA, short-acting muscarin antagonist; SAT, blood oxygen saturation; WBC, white blood cell count.

## Data Availability

The data upon which this analysis was based are available from Professor Hans Lennart Persson in anonymized form upon receipt of a reasonable request.
